# The link between climate change, food security and fertility: The case of Bangladesh

**DOI:** 10.1371/journal.pone.0258196

**Published:** 2021-10-21

**Authors:** Mengni Chen, Shah Md Atiqul Haq, Khandaker Jafor Ahmed, A. H. M. Belayeth Hussain, Mufti Nadimul Quamar Ahmed

**Affiliations:** 1 Department of Sociology, Copenhagen University, Copenhagen, Denmark; 2 Centre for Demographic Research, Université Catholique de Louvain, Louvain, Belgium; 3 National Fund for Scientific Research, Louvain, Belgium; 4 Department of Sociology, Shahjalal University of Science and Technology, Sylhet, Bangladesh; 5 Department of Geography, Environment and Population, The University of Adelaide, Adelaide, South Australia, Australia; 6 School of Social Sciences, University Sains Malasia (USM), Penang, Malaysia; 7 Department of Applied Sociology and Social Work, North East University Bangladesh, Sylhet, Bangladesh; Neijiang Normal University, CHINA

## Abstract

Climate change is likely to worsen the food security situation through its impact on food production, which may indirectly affect fertility behaviour. This study examines the direct and indirect effects of climate change (e.g., temperature and precipitation) via the production of major crops, as well as their short- and long-term effects on the total fertility rate (TFR) in Bangladesh. We used structural equation modelling (SEM) to perform path analysis and distinguish the direct influence of climate change on fertility and its indirect influence on fertility through food security. We also applied the error correction model (ECM) to analyze the time-series data on temperature and precipitation, crop production and fertility rate of Bangladesh from 1966 to 2015. The results show that maximum temperature has a direct effect and indirect negative effect–via crop production–on TFR, while crop production has a direct positive effect and indirect negative effect–via infant mortality–on TFR. In the short term, TFR responds negatively to the maximum temperature but positively in the long term. The effect of rainfall on TFR is found to be direct, positive, but mainly short-term. Although indicators of economic development play an important part in the fertility decline in Bangladesh, some climate change parameters and crop production are non-negligible factors.

## Introduction

Climate change is a major problem today and poses a threat to the entire world. The Earth’s atmosphere’s average temperature has increased by about 0.15°C to 0.2°C over the past 100 years [1]. By the end of this century, global temperatures are expected to increase by 0.3°C to 4.8°C [2]. Precipitation patterns have also changed globally and locally [3]. There are positive and negative trends in precipitation worldwide. The trend is increasing in tropical seas and decreasing in some areas at mean altitudes [4]. However, seasonal variations in precipitation–particularly in much of the arid tropics–show the increasing uncertainty in intensity, timing, and duration [5]. In addition to climate change, the world experienced more than 11,000 extreme weather events between 1997 and 2016, resulting in more than 524,000 deaths and $3.16 trillion in economic losses. According to the 2020 Global Climate Risk Index (CRI), the top ten countries most affected by the number of extreme events between 1999 and 2018 are: Philippines (n = 317), Vietnam (n = 226), Bangladesh (n = 191), Nepal (n = 180), Pakistan (n = 152), Thailand (n = 147), Haiti (n = 78), Myanmar (n = 55), Puerto Rico (n = 25), and Dominica (n = 8) [6].

About 108 million people living in 48 countries at risk of food crises were highly vulnerable or already experiencing acute food insecurity in 2016 [7]. Climate change reduces agricultural production and increases food insecurity due to abnormal precipitation and temperature changes [8–10]. The impact of climate on agriculture is region-specific mainly [11]. Bangladesh is one of the most vulnerable countries to climate change and variability [12, 13]. According to the Global Climate Risk Index 2020, Bangladesh experienced 191 climate-related extreme events between 1999 and 2018 [6]. Extreme climate events such as floods, cyclones, and droughts are common in Bangladesh [14–16], which have serious impacts on the livelihoods of vulnerable populations [17–19]. Climate change, such as the steady rise in temperature and changes in precipitation patterns, has severely affected several sectors in Bangladesh, including agriculture, water resources, and public health [18, 20]. The cultivation of crops, especially rice, is often limited by various climate hazards–floods, droughts, soil and water salinity, cyclones, water waves, etc.–in the country.

Population dynamics such as mortality, migration, and fertility can undergo significant changes due to climate variability or natural disasters [21–23]. Human geographers and demographers have previously studied the effects of climate variability on health [24, 25], migration [26–28], and mortality [29]. Climatic extremes have already adverse effects on Bangladesh’s population sectors, including child mortality [30], migration [31], and child marriage [32–34]. In this study, we are interested in the effect of climate change on fertility, which has received less attention [35]. There are studies on the relationship between environment and ideal family size and family planning practices [36–43]. Thus, poor environmental conditions or increasing environmental degradation are expected to affect child demand and fertility. The effect of firewood and water scarcity on fertility was found to be positive in South Africa [36] and Nepal [39], while another study in Nepal found a negative relationship [44]. Similarly, a positive relationship between water scarcity and fertility was found in Honduras and Nepal [38]. In response to fuel and water shortages and increased harvest seasons, married indigenous men and women living in reserved forests in Bangladesh were more likely to have more children who could work and earn money through other livelihood opportunities [41]. In terms of the effect on family planning behaviour, Ghimire and Mohai found that, in Nepal, those who believed that current agricultural productivity had decreased from three years earlier were more likely to use contraceptives than those who believed that current agricultural productivity had remained about the same or increased [40].

Since Bangladesh is a predominantly agricultural country, climate change could exacerbate food insecurity by affecting crop production and, indirectly, fertility. This study aims to find out if and how climate change affects fertility in the country. Specifically, the direct and indirect effects of climate change (e.g., temperature and precipitation) on major crops and the short- and long-term effects on fertility are examined. The results of this study can add exciting insights and arguments to the existing literature on population dynamics and climate change impacts. They can also deepen our understanding of the linkages between climate change, food security, and fertility.

The next section presents the conceptual framework of this study in relation to climate change, food security, and fertility to justify and explain the purpose of the study. This is followed by a description of the data and methodology used in the analysis. Then, the results regarding the impact of climate change and crop production on fertility are presented. Finally, a concluding section discusses the implications of the main findings and provides recommendations for further research.

## Conceptual framework

### Impacts of climate change on food security

Crop production in South Asian countries is likely to be most affected by climate change. For example, a temperature increase between 1.0°C and 3.0°C could reduce rice production in Punjab by 3–10% [45]. In Tamil Nadu, rice production decreased by up to 41% in response to a 4°C temperature increase [46]. In Pakistan, Ali et al. found that maximum temperature was negatively associated with wheat production, while the effect of minimum temperature was positive and significant for all crops (wheat, rice, maize, and sugarcane). Moreover, the effect of rainfall on crop yield was negative except for wheat [47]. Esham et al. also found in Sri Lanka that temperature is negatively associated with maize yield, while rainfall is positively associated with maize yield [48]. In Indonesia, Levine and Yang found that more rainfall in a year increases rice production [49]. This literature suggests that temperature has adverse effects and rainfall has positive effects on South Asian countries’ major crop production, indicating that their primary crop production is highly susceptible to climate change and variability.

In Bangladesh, agriculture absorbs about 44% of the workforce [50]. 76% of population live in rural areas, and 90% of them are directly involved in agriculture. The net crop income is sensitive to climate changes [51]. Despite technological advancement, the climate still plays a crucial role in agriculture, where temperature and rainfall are the key determinants of crop production [52]. In this study, the analysis includes the most three major crops- rice, wheat, and pulses. As for rice, the climatic impact varies across different types of rice [53–55]. Sarker et al. evaluated the effects of climate change (e.g., temperature and precipitation) for three major rice crops (Aus, Aman, and Boro) in Bangladesh and found significant effects of climate change on rice yield. Their findings revealed that maximum temperature has positive effects on Aus and Aman rice and negative on Boro; the minimum temperature has adverse effects on Aman and positive on Boro; rainfall has positive effects on Aus and Aman [53]. The production of wheat is susceptible to temperature in Bangladesh [56, 57].

### Links between climate change, food security and fertility

Climate change, such as changes in precipitation patterns and frequent temperature fluctuations, can affect population dynamics through fertility behaviour [35, 58]. There are some recent studies on how fertility rate or outcome responds to climate shocks [59–62]. For example, higher temperatures reduce birth rates about nine months later [59]. Lam and Miron found that a 1°C increase in monthly temperature would lead to nearly 1% fewer births nine months later for the white population (62). Similarly, a 1°C increase in summer temperature leads to 1% [63], 0.5%, or more [64] fewer births nine months later in many U.S. states.

Not only temperature but also precipitation is related to fertility [62]. A study of rural communities in Mexico found that fertility–in the years following an increase in precipitation–was 1.14 times higher for dryland communities than for wetland communities. This implies that the previous year’s precipitation is an important factor in fertility timing, especially for dryland communities. The author explains that during a drought period in arid areas, households may prefer short-term migration to find work in the absence of livelihood opportunities. Once environmental conditions improve after above-average rainfall, household members were likely to stay home and work on the farm rather than migrate [62]. In Bangladesh, dry spells were associated with increased migration of male household heads, while farmers were 40% more likely to migrate after a dry spell compared to their peers engaged in other livelihoods [31]. Sellers and Gray revealed that the intention to have another child increased and the use of family planning decreased in response to delays in the monsoon in the previous year, especially among wealthier and more educated women. As a result of abnormally high temperatures in the past five years, women with farms were less likely to give birth because they made greater use of family planning, especially among poorer and less educated women [61].

The short- and long-term effects of climate variability on fertility can be different. A recent study conducted in 18 sub-Saharan African countries shed light on the short- and long-term effects of climate variability on fertility. In the study, Eissler et al. found that temperature increase, both short- and long-term, has negative effects on fertility, while precipitation, both short- and long-term, has differential effects on fertility. For example, a one standard deviation increase in temperature for 12 months (short term) and 60 months (long term) leads to a 0.042 and 0.101 reduction in ideal family size, respectively. In contrast, a one standard deviation increase in precipitation is associated with a 0.052 decrease in children in the short term, while a one standard deviation increase in precipitation is associated with a 0.059 increase in women’s preference for ideal family size in the long term [65].

The effects of climate variability may have indirect effects on fertility through food production. Food insecurity leads to a range of demographic impacts, including but are not limited to changes in fertility. Reducing human fertility–a means of controlling population growth–may be one way to cope with inadequate food supply [66]. Changes in fertility due to food insecurity are usually explained by the effects of drought and associated famine. However, a study in sub-Saharan Africa found that fertility rates were lower among populations enjoying food security than among populations not enjoying food security [67]. Fertility rates were found to decline after drought and associated famine. For example, there was a significant decline in conception rates in Ethiopia during the years of drought and famine between 1970 and 1980 [68]. Similarly, fewer conceptions were reported during the months of January through May, when there were extreme food shortages in Finland due to the 1967–68 famine [69]. The historic famine in China from 1958 to 1961 had a similar effect on fertility [70, 71]. A study of the effects of the 1974 famine in Bangladesh showed that the impact on fertility varied from lower to higher socioeconomic groups. The separation of couples explains the pronounced negative effect on lower socioeconomic groups as spouses migrated to other regions of the country searching for sources of income, which affected the frequency of sexual intercourse [72].

Drawing on insurance mechanisms and replacement theory, Finlay argues that fertility responds to both natural disasters and infant mortality [73]. In a study in India, Pakistan, and Turkey, Finlay concludes that people have a “positive response” to child mortality [73]; people who have lost children in disasters prefer replacement [74, 75] and view more children as insurance against expected risk [74, 76]. In Indonesia, women who had lost one or more children during the disaster were more likely to plan to have more children after the tsunami. Before the tsunami, women who did not have children “were more likely to start families in communities where tsunami-related mortality rates were higher” [74]. Lindstrom and Kiros examined short- and long-term fertility responses to infant and child mortality in Ethiopia. They found that fertility responses tend to be high among people who have experienced infant mortality, which is referred to as replacement behaviour or replacement effect [77]. Owoo et al. conducted a study in Ghana and found that women living in a place with higher mortality shocks tended to have higher fertility preference [78].

The literature section shows that Bangladesh’s economy is largely dependent on agriculture, which is extremely vulnerable to climate change. The literature also shows that the global impacts of climate change on crop production and the resulting food insecurity are visible and studied in different countries. In response to food insecurity, vulnerable populations see demographic adaptations, such as changes in fertility behaviour, as coping mechanisms. Research has already examined the effects of climate variables on crop production and fertility changes in different areas separately. However, to the best of our knowledge, there is no such attempt made to jointly study the direct and indirect, as well as short- and long-term, effects of climate variables on crop production and subsequently fertility.

## Data and methodology

This study incorporates climate change, production of major crops (i.e., rice, wheat, and pulses), and fertility data of Bangladesh during 1966–2015. We collected climate data from the Bangladesh Meteorological Department (BMD) (http://www.bmd.gov.bd) [79]. Then we calculated the annual maximum, average and minimum values of temperature and precipitation for the period 1966–2015. Since the minimum temperature and precipitation for some years are zero, we did not use the two indicators for the convenience of our analysis. We also collected annual data for rice, wheat, and legume production from the United Nations Food and Agriculture Organization (FAO) (http://www.fao.org/home/en/) [80]. The data for infant mortality and total fertility rate (TFR) was from the United Nations Population Divisions (https://population.un.org/wpp/) [81]. Because crop production is not only sensitive to climatic factors but also to fertilizer use, we include fertilizer consumption (i.e., the amount of plant nutrients used per unit of arable land) as a control variable, with data from the World Bank (https://data.worldbank.org/). The dependent variable is TFR. The independent variables are temperature, rainfall, infant mortality, as well as rice, wheat, and legume production. The GDP per capita, which reflects the average income of the population, is also included as a control variable. However, because crop production is an important factor of agriculture income and a crucial contributor to the GDP in Bangladesh, GDP per capita becomes an endogenous factor. To avoid endogeneity of GDP per capita, we finally decided to take the GDP per capita from the industry and service sector, that is, GDP per capita*(1 - % of agriculture, forest and fishing value add in GDP). Both the GDP per capita and the percent of agriculture, forest and fishing value add in GDP are from the World Bank (https://data.worldbank.org/). For simplicity, in the remaining paper, we use the term “GDP per capita” to represent “the GDP per capita from the industry and service sector”.

To examine the direct and indirect impact of climate change, we adopt the structural equation model (SEM) to perform pathway analysis. Path analysis allows us to distinguish the direct impact of climate change on fertility and its indirect impact on fertility through food security. To assess the short- and long-term relationship between climate change and fertility, we apply the error correction model (ECM). ECM is a very useful econometric method for exploring time series data, which allows us to study the long-term equilibrium relationship between climate change and fertility, as well as how fertility responds to climate shock in the short term. Because agricultural production and infant mortality are endogenous, we do not include them in the ECM model. The error-correction equations are formulated as

$$\Delta TFR=\sigma +\sum_{i=1}^{p}\gamma_{j}\Delta {TFR}_{t-i}+\sum_{j=1}^{p}\eta_{j}\Delta {climate}_{t-j}+\sum_{m=1}^{p}\xi_{m}\Delta {GDPPC}_{t-m}+\lambda {ECT}_{t-1}+u_{t}$$ (1)

$${ECT}_{t-1}={TFR}_{t-1}-\alpha {climate}_{t-1}-{\beta GDPPC}_{t-1}$$ (2)

In the equations above, TFR, climatic variables and GDP per capita are measured in logs. Δ denotes first differences. *σ* refers to the constant. *γ_j_*, *η_j_*, *ξ_m_* are the parameters that denote the short-term elasticities. In Eq (1), *ECT*_*t*−1_ refers to the error correction term, representing the long-term relationship among the TFR, climatic indicator, and GDP per capita. *λ* is the adjustment parameter representing the speed of convergence to the equilibrium path. The error correction term is specified in Eq (2). *α* and *β* reflects the long-term relationship of TFR with respect to climatic indicators and GDP per capita. We first test the variables for their unit-roots to check their stationarity through the Augmented Dickey-Fuller test. Second, we test for cointegration using the Johansen test. Finally, we run ECM models. We use the software package of STATA 16 for path analysis and ECM modelling.

## Results

### Descriptive statistics

**Fig 1:**
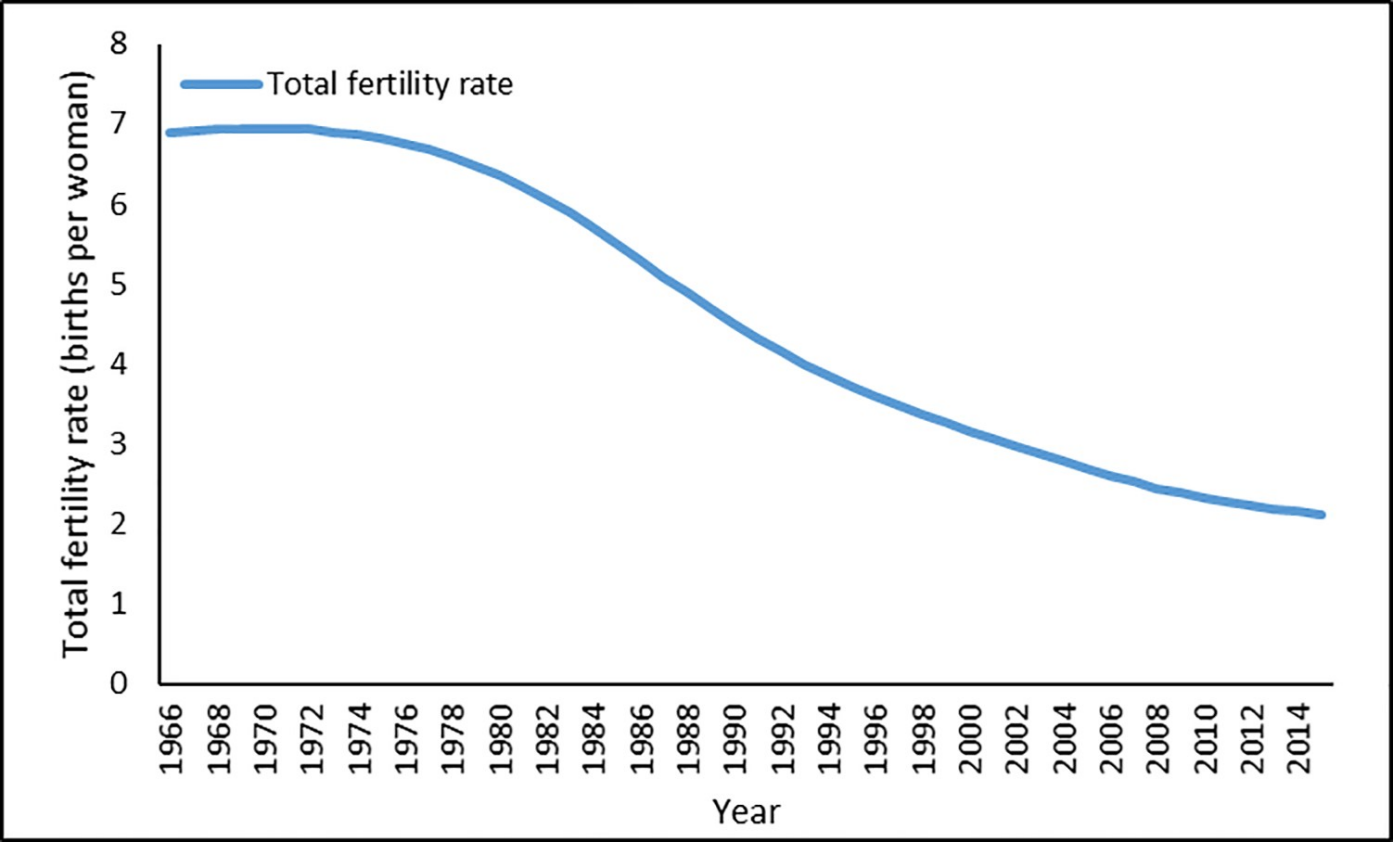
Total fertility rate in Bangladesh for 1966–2015.

**Fig 2:**
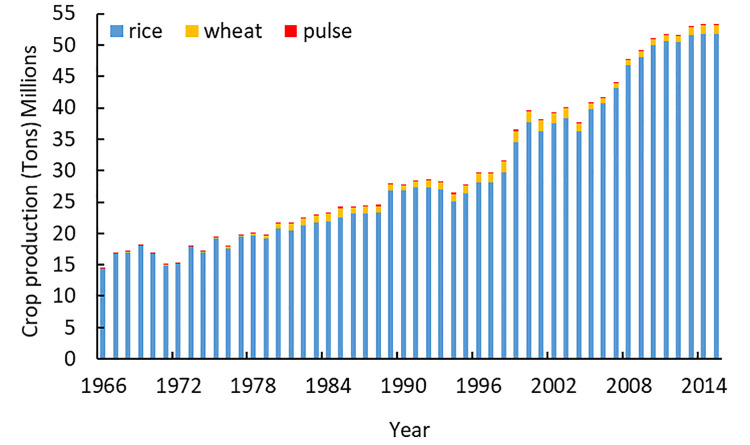
Major crop production in Bangladesh for 1966–2015.

Bangladesh’s fertility trends from 1966–2015 are shown in Fig 1. The rapid decline of fertility in the last five decades in Bangladesh is, indeed, a historic record in the demographic transition. TFR declined from around 7 (children per woman) in 1966 to 2.1 (children per woman)–the replacement level–in 2015. Although the extent and rapidity of the fertility decline have been very impressive, the pace of decline could be influenced by climate change and its adverse effects on food production in Bangladesh. Fig 2 shows agricultural production over time. Over this period, production of the main crops–rice, wheat, and pulses–in Bangladesh has been increasing, except in 1971, 1972, 1974, 1976, 1994, and 2004. The reduction in crop production in these years could be attributed to the effects of war in 1971–1972, the effects of drought in 1974 and 1976, and crop losses due to flooding in 1994 and 2004, respectively. Despite the increase in food production with the advanced use of technology, Bangladesh still remains food insecure, which could be exacerbated by climate variability changes.

**Fig 3:**
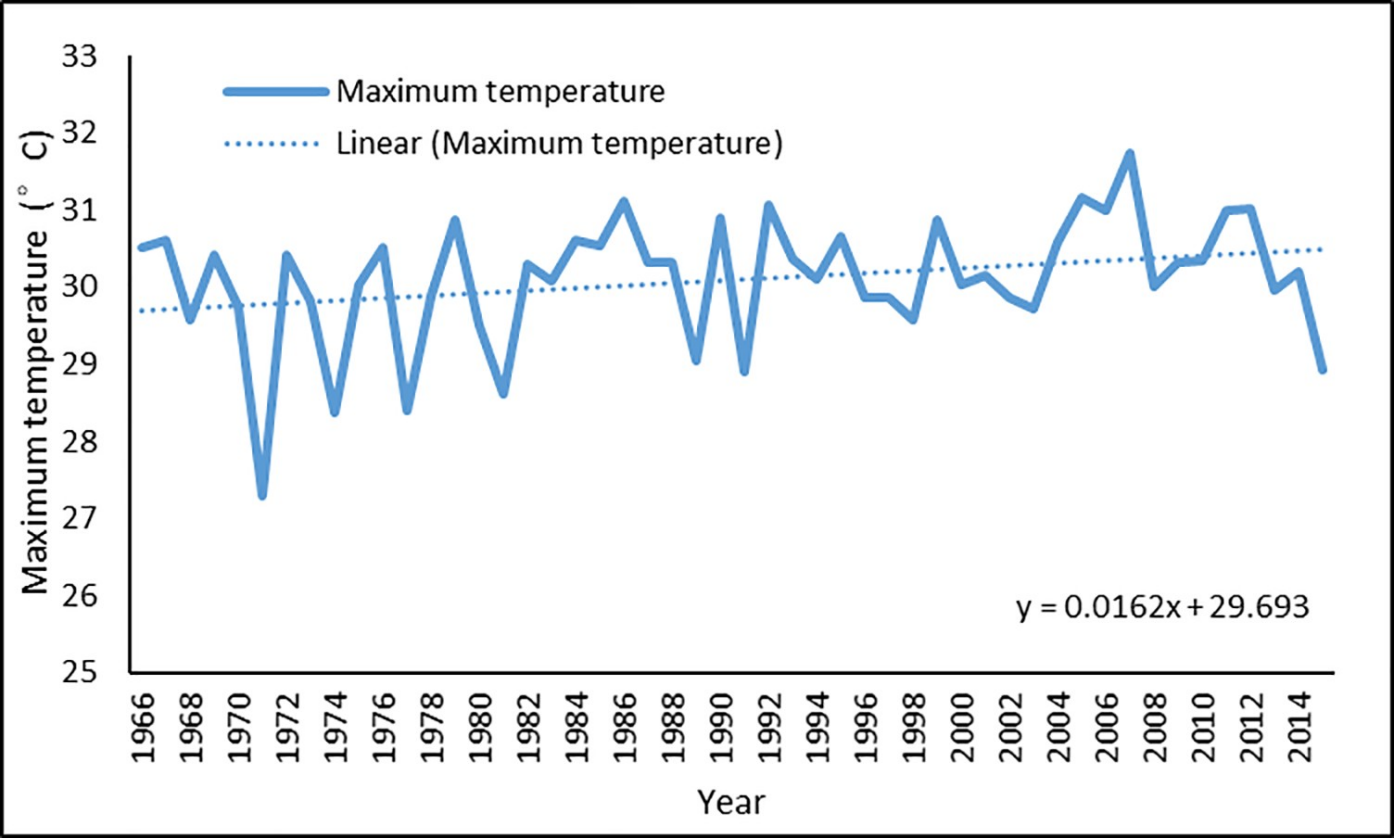
Annual average of maximum temperature in Bangladesh for 1966–2015.

**Fig 4:**
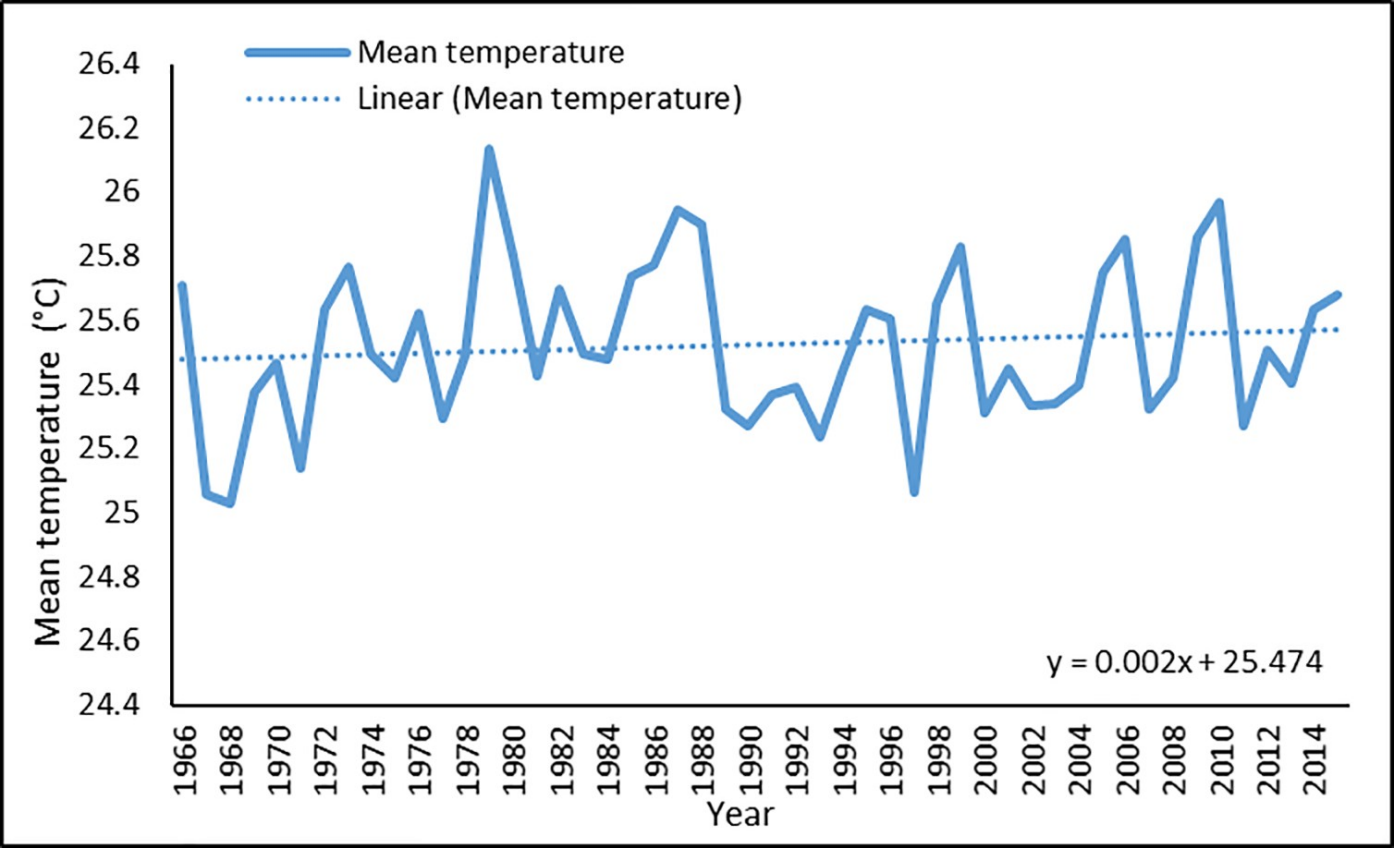
Annual average of mean temperature in Bangladesh for 1966–2015.

**Fig 5:**
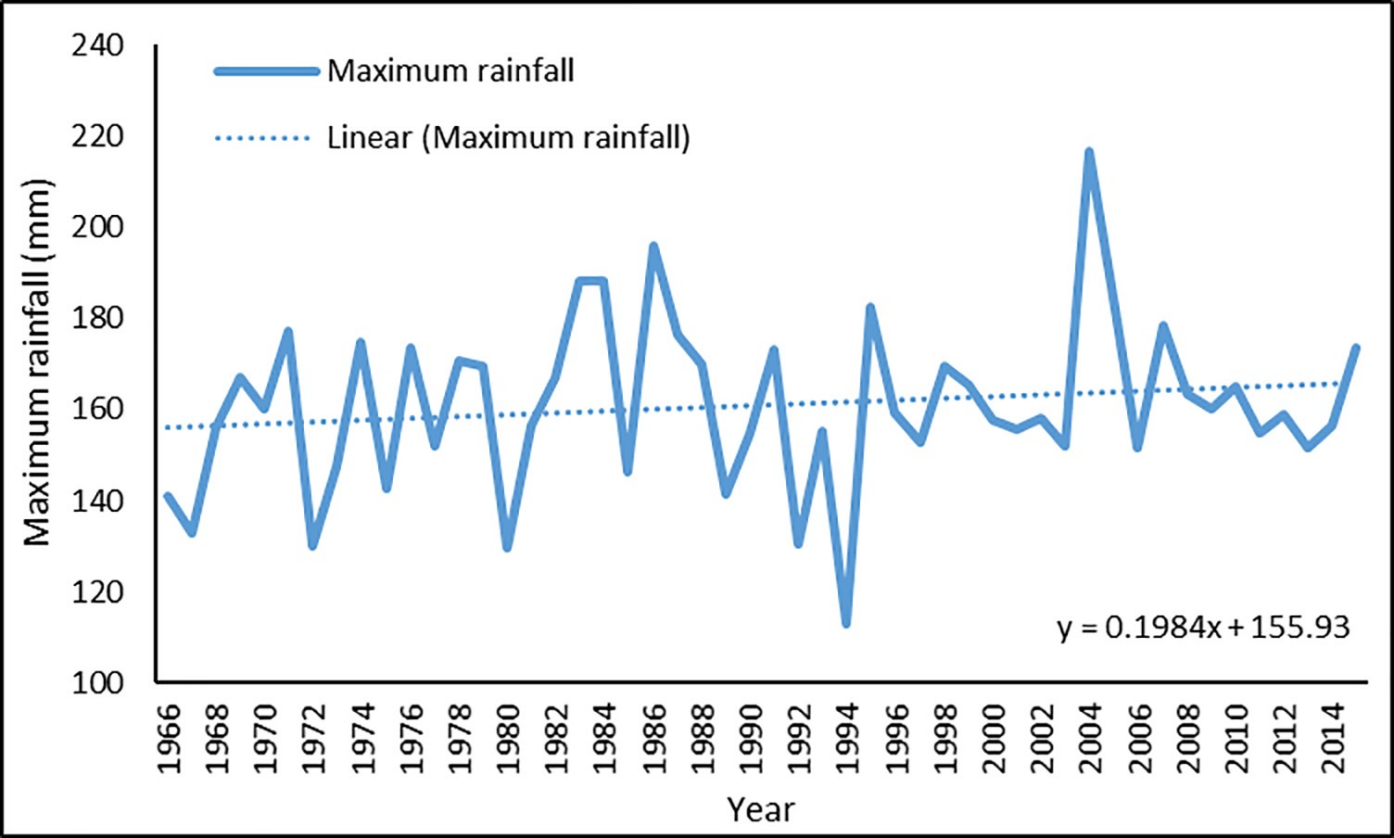
Annual average of maximum rainfall in Bangladesh for 1966–2015.

**Fig 6:**
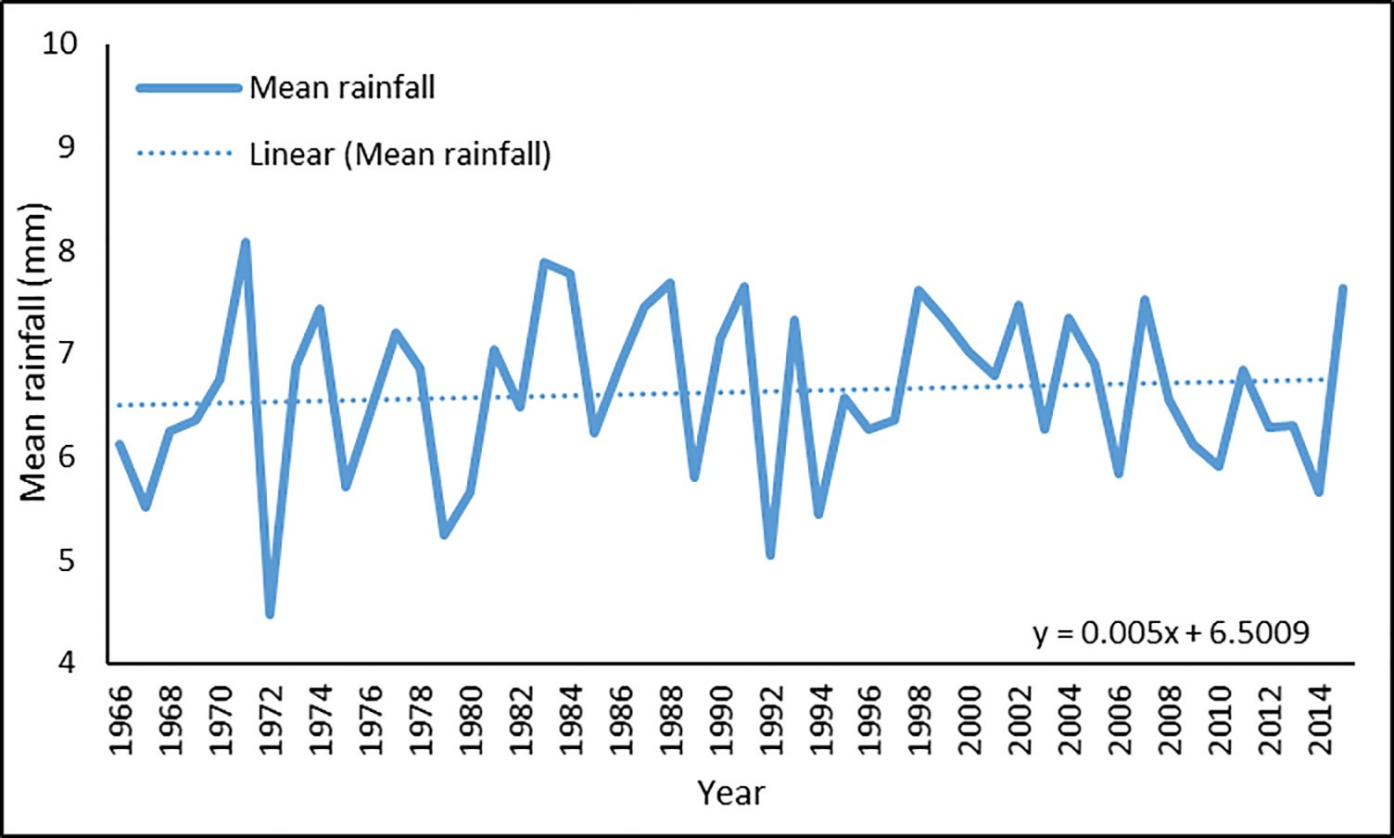
Annual average of mean rainfall in Bangladesh for 1966–2015.

Figs 3 and 4 show the annual average maximum and mean temperature, respectively. Although highly fluctuating, the temperature has followed a slight upward trend. A linear estimate shows that the maximum temperature increased at a rate of 0.016 degrees Celsius per year during the five decades, while the mean temperature increased by 0.002 degrees Celsius each year. Figs 5 and 6 show the annual average maximum and mean daily precipitation, respectively. Precipitation also shows a slightly increasing trend with fluctuation over this period. According to the linear estimate, the maximum daily rainfall increased by 0.198 mm per year, while the average daily rainfall increased by 0.005 mm.

### Direct and indirect impacts of climate change on TFR

**Fig 7:**
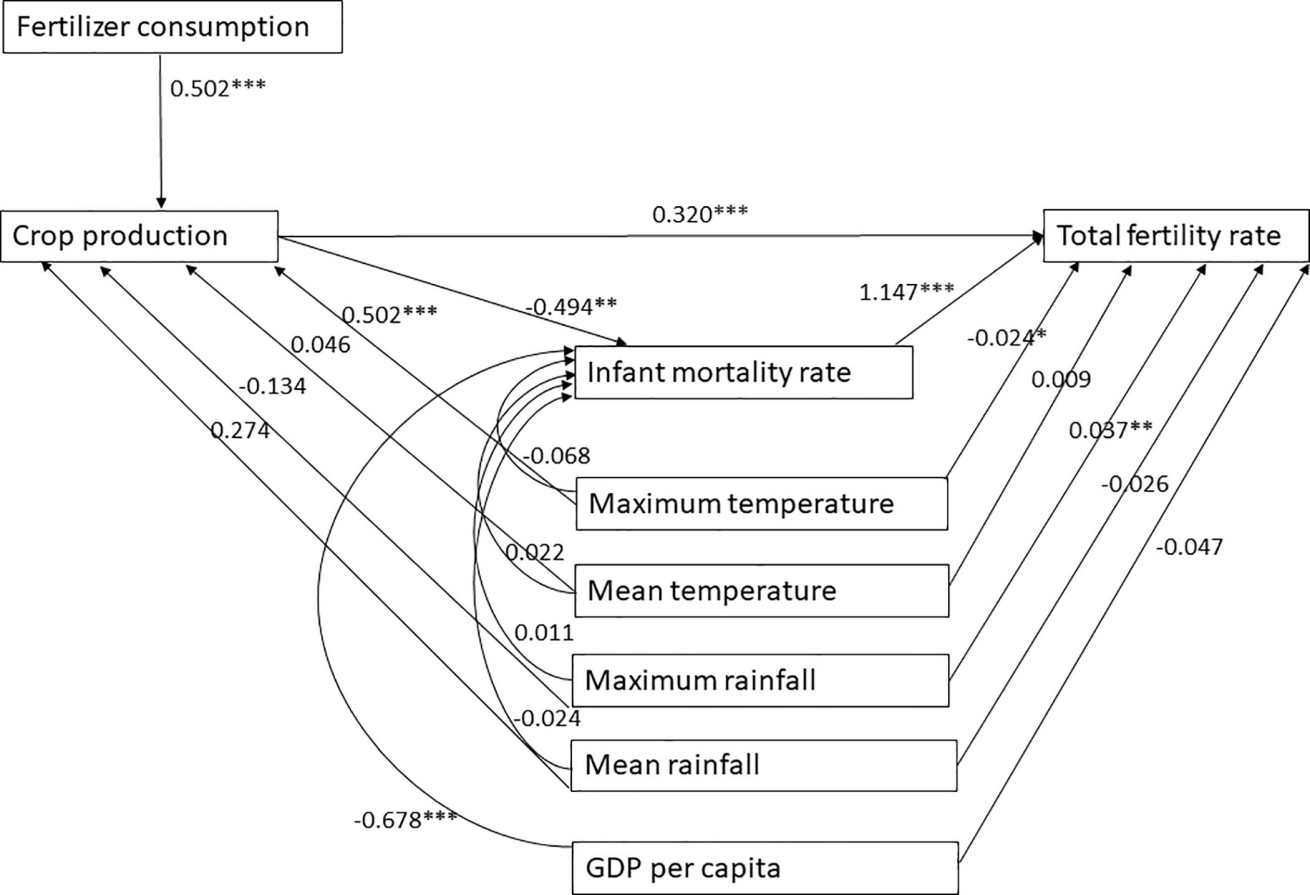
The path analysis.

The standardized path coefficients from the path analysis are shown in Fig 7. Fertilizer consumption and maximum temperature have a significant positive impact on crop production. Crop production has a direct positive impact on TFR and an indirect impact on TFR through a negative impact on the infant mortality rate. And the infant mortality rate has a positive impact on the TFR. The maximum temperature has a significant direct negative effect, while maximum rainfall has a significant direct positive effect on the TFR. For example, if maximum temperature increases by one standard deviation of its mean values, the TFR will decrease by -0.02 standard deviation of its mean values; if maximum rainfall increases by one standard deviation, the TFR will increase by 0.032 standard deviations. Besides, GDP per capita has a negative effect on the infant mortality rate, thus reducing the TFR indirectly. The direct effect of GDP per capita on the TFR is not statistically significant. In summary, Fig 7 suggests that although economic development indicators such as crop production and infant mortality are critical in shaping fertility transition in Bangladesh, the maximum temperature and rainfall have a direct impact on the TFR and maximum temperature also indirectly affects crop production, while crop production has a direct and an indirect impact on the TFR through the infant mortality rate.

The direct, indirect, and total effects of all factors are shown in Table 1. The total effect of maximum temperature on TFR is significantly negative, mainly due to the direct negative effect. Regarding maximum rainfall, despite its significant direct positive impact on TFR, the total impact is insignificant. Meanwhile, the mean temperature and rainfall have no significant direct, indirect, or total effect on TFR. The total effect of crop production is negative but insignificant, and this is because the negative indirect effect offsets the positive direct effect. The direct impact of infant mortality on TFR is significantly positive. Lastly, the GDP per capita has a significant negative indirect effect through the infant mortality rate, thus leading to a significant negative total effect on TFR.

**Table 1:** Direct, indirect, and total effects of selected factors on TFR.

Variables	Direct effect	Indirect effect	Total effect
Maximum temperature	-0.045*	-0.387	-0.432*
Mean temperature	0.056	0.086	0.142
Maximum rainfall	0.003**	0.004	0.007
Mean rainfall	-0.050	-0.187	-0.237
Crop production	0.042***	-0.074**	-0.032
Log(GDP per capita)	-0.144	-2.368***	-2.512**
Infant mortality	0.052***	/	0.052***
Fertilizer	/	-0.008	-0.008***

Note

* p<0.05

** p<0.01

*** p<0.001.

### Short-term and long-term impacts of climate change on TFR

Furthermore, we perform ECM modelling to estimate the short-term and long-term effects of climatic factors. Table 2 shows the result of the unit root test. As shown, not all variables are stationary in their log level (e.g., GDP per capita), while they are all stationary at 5% when using the first-differenced data. Therefore, the undifferentiated variables are used for the cointegration test, and the first-differenced variable was used for the ECM model. Because the crop production and infant mortality are endogenous, we do not include them in the ECM model. Four cointegration vectors are preferred by Johansen’s test for the variable sets "ln(TFR), ln(maxtemp), ln(meantemp), ln(maxrain), ln(meanrain), ln(GDPPC)".

**Table 2:** Unit-root test.

Variables	Definition	Z(t)	5% critical values
ln(TFR)	log of total fertility rate	-4.079	-2.938
Δln(TFR)	log of first difference of total fertility rate	-4.319	-3.628
ln(GDP per capita)	log of GDP per capita	1.202	-2.938
Δln(GDP per capita)	log of fist difference of GDP per capita	-3.080	-2.941
ln(maxtemp)	log of maximum temperature	-2.695	-2.938
Δln(maxtemp)	log of first difference in maximum temperature	-6.043	-2.941
ln(meantemp)	log of mean temperature	-3.523	-2.938
Δln(meantemp)	log of first difference of mean temperature	-6.060	-2.941
ln(maxrain)	log of maximum rainfall	-3.905	2.938
Δln(maxrain)	log of first difference of maximum rainfall	-6.124	-2.941
ln(meanrain)	log of mean rainfall	-3.844	-2.938
Δln(meanrain)	log of first difference in mean rainfall	-7.084	-2.941

Table 3 shows the results from ECM analysis. The ECM model has two parts of the results. One part is about the short-term impact, and the other part–the error correction term–is about the long-term impact in an equilibrium situation. It should be noted that when interpreting the error correction term, all coefficients should be inverted.

**Table 3:** The result of ECM modelling.

	Model 1
**Short term**	
λ	-0.007***
Δln(TFR_t-1_)	0.591***
Δln(TFR_t-2_)	0.349*
Δln(maxtemp_t-1_)	-0.074***
Δln(maxtemp_t-2_)	-0.048**
Δln(meantemp_t-1_)	0.036
Δln(meantemp_t-2_)	0.058
Δln(maxrain_t-1_)	0.007
Δln(maxrain_t-2_)	0.003
Δln(meanrain_t-1_)	0.010**
Δln(meanrain_t-2_)	0.006
Δln(GDPPC_t-1_)	0.002
Δln(GDPPC_t-2_)	-0.012*
**Error correction term (long term)**	
ln(TFR)	1
ln(maxtemp)	-7.39**
ln(meantemp)	10.784
ln(maxrain)	1.061
ln(meanrain)	1.290
ln(GDPPC)	0.499***

Note: the coefficients in the error correction term should be interpreted in the opposite direction.

* p<0.05

** p<0.01

*** p<0.001.

In model 1, the value of λ is -0.007. Statistically, it means that once the vector of TFR, GDP per capita, and the four climate factors deviate from the long-term equilibrium path, it will converge to equilibrium at a rate of 0.007. In the short term, the change in maximum temperature has a significant negative impact on the change in TFR, with a coefficient of -0.074 for the first lagged year and -0.048 for the second lagged year. This means that if the maximum temperature increases by 1%, the TFR will decrease by approximately 0.074% one year later and by 0.048% two years later. Moreover, the change in the mean rainfall has a positive impact on the change in TFR. In addition, we find that fertility responds negatively to GDP per capita in the short term.

The error correction term reveals a long-term relationship between fertility, GDP per capita, maximum temperature, mean temperature, maximum rainfall, and mean rainfall. Only the coefficients of ln(maxtemp) and ln(GDPPC) are significant, with values -7.39 and 0.499, respectively. This means that in the long-term equilibrium, the increase in maximum temperature would have a positive impact on the TFR and the increase in GDP per capita would have a negative impact on the TFR.

## Concluding discussion

Path analysis shows that climatic factors, especially maximum temperature and precipitation, can affect fertility directly and to some extent only maximum temperature indirectly through crop production, while crop production affects fertility directly and indirectly through infant mortality. But the contribution of indicators of economic development is also crucial in influencing fertility rates directly or indirectly. Higher GDP per capita is likely to reduce fertility rates by reducing infant mortality.

Indirectly speaking, the increasing maximum temperature is likely to increase crop production; increasing crop production is likely to reduce infant mortality; decreasing infant mortality contributes to the fertility decline. The positive relationship between maximum temperature and crop production is consistent with the findings from the study by Sarker et al., who also found a positive association of maximum temperature with Aus and Aman variety in Bangladesh [53]. Crop production has a significant negative impact on the infant mortality rate. Increased food production increases food security, ensures food intake and reduces susceptibility to diseases [82], improving women’s reproductive health and increasing child survival. In other words, increased food insecurity is directly associated with increased child mortality [83, 84]. Our path analysis confirms that the infant mortality rate positively impacts the TFR, which is consistent with the literature and existing theories of the replacement effect [74, 75, 85, 86].

Different from findings in Winkler-Dworak’s study has that the context of sub-Saharan Africa [67], fertility decreases as food production increases, we have demonstrated a positive direct effect of crop production and a negative indirect effect of crop production (via infant mortality) on the fertility, thus resulting in a significant total impact. Moreover, the total effect of maximum temperature on TFR is significantly negative, mainly because of the negative direct effect. It means that as maximum temperature increases, TFR decreases. This is consistent with previous studies that warm weather reduces birth rates nine months later [63, 64, 87].

In assessing the short- and long-term relationship between climate change and fertility, we ran error correction models. Fertility is found to respond negatively to maximum temperature in the short run but positively in the long run. Eissler et al. found that both short- and the long-term temperature has adverse effects on fertility [65]. The inconsistency with the findings of Eissler et al. [65] is likely because their study focuses on sub-Saharan African countries, where the climate is very different from Bangladesh. The reduction in fertility with increasing short-term maximum temperature in our study could be explained by increased migration, which in turn could affect the frequency of sexual intercourse. Carrico and Donato conclude that extreme weather conditions such as drought periods in Bangladesh are more consistently associated with increased migration of male household heads [31]. In addition, reduced birth rates could also be attributed to worse reproductive health at conception [59]. Besides, although fertility responds positively to the mean rainfall in the short term, it is insensitive to the long term. This seems to contradict the results of a recent study by Eissler et al. in 18 Sub-Saharan African (SSA) countries that long-term (60-month) increases in rainfall are associated with an increase in women’s preference for fertility [65].

While our results confirm that GDP per capita is largely responsible for fertility decline in both the short and long run, we show that certain climate change parameters (e.g., maximum temperature and precipitation) and crop production are also non-negligible factors for fertility transition in the country. Although GDP per capita is responsible for fertility decline in both the short and long term, we show that climate change and crop production are also non-negligible factors for fertility transition in the country. This study can serve as an example and focus further research on regions or countries that are in a similar context to Bangladesh, whose economies are mainly based on agriculture and are highly vulnerable to climate change. Climate change is likely to worsen a country’s economic development and may affect fertility rates directly or indirectly, in the short or long term. Since GDP per capita is one of the determining factors for the decline in fertility rate in Bangladesh, the adverse effects of climate change may affect the rate of this decline by reducing GDP growth. The study findings advance our understanding of the impacts of climate change parameters with an indirect impact through food production on the fertility rate in Bangladesh. This study suggests further research incorporating multiple climate change parameters and crop types with a particular focus on spatial variations across the districts of Bangladesh. Further research could separate the effects of different crops in the analysis.

Since crop production (for example, rice and wheat) is susceptible to temperature in Bangladesh, variety-specific adaptation strategies could help mitigate the adverse effects of climate change and thereby reduce the risk of food insecurity. In Bangladesh, several salt-tolerant high-yielding rice varieties have been successfully released to coastal areas in recent years, which could ensure food security for vulnerable coastal populations [88]. Other areas vulnerable to various extreme weather events such as floods, droughts, extreme temperature events etc., need immediate attention. Policymakers should build the adaptive capacity of populations vulnerable to climate change by increasing the storage of crops needed for times of crisis and providing financial support to help recover from the impacts of climate-related events and loss of agricultural production.

There are some limitations to this study. First, TFR is used as a measure of fertility, which refers to the average number of children per woman in a given year. It is not possible to distinguish whether the change in the TFR is due to the change in the number of children desired by women or to the change in the state of health of the women who give birth or in fertility. Second, the current analysis is nationwide. Having no access to provincial and county-level data, we are unable to reveal whether the relationship between fertility and climate factors varies from one region to another. Third, this study only considered infant mortality, agricultural production and climate factors in the analysis. We are aware that there are many other agricultural inputs (e.g. agricultural technology, irrigated land, etc.) and various fertility factors (e.g. education, labour force participation, etc.); however, due to data limitation, we cannot incorporate these factors into the analysis.

## Supporting information

S1 Data (XLSX)
